# 

**Published:** 1931

**Authors:** 


### CONTENTS.

| | Page |
|---|---|
| **Some Reminiscences and a Forecast.** By SIR EWEN J. MACLEAN, M.D., D.Sc. (Hon.), F.R.C.P., F.A.C.S. (Hon.), F.R.S.E. .. .. | 83 |
| **The Modern Outlook on Mental Health.** By L. G. BROCK, C.B. | 97 |
| **Some Intestinal Malformations and their Clinical Significance.** By ARTHUR L. TAYLOR, M.D. .. .. .. | 113 |
| **Some Disorders of Movement and their Mechanism.** By H. H. CARLETON, M.D., M.R.C.P. .. .. .. .. .. | 135 |
| **Strangulated Hernia.** By NOËL KEMM, M.B., B.S., F.R.C.S. .. | 151 |
| **Reviews of Books** .. .. .. .. .. .. .. .. | 155 |
| **Editorial Notes** .. .. .. .. .. .. .. .. | 159 |
| **Meetings of Societies** .. .. .. .. .. .. .. | 162 |
| **The Medical Library of the University of Bristol** .. .. | 164 |
| **Local Medical Notes** .. .. .. .. .. .. .. .. | 165 |

